# Localized skin‐limited blastic plasmacytoid dendritic cell neoplasm

**DOI:** 10.1002/jha2.383

**Published:** 2022-03-01

**Authors:** Edwin U. Suárez, Javier Cornago, Miguel Á. Piris, Socorro M. Rodriguez Pinilla, José L. López‐Lorenzo, Carlos Soto

**Affiliations:** ^1^ Department of Haematology Hospital Universitario Fundación Jiménez Díaz Madrid Spain; ^2^ Department of Pathology Hospital Universitario Fundación Jiménez Díaz Madrid Spain

A 32‐year‐old man presented with a 7‐month history of progressive painless, nonpruritic skin and soft tissue lesion on his left leg. He had no systemic or B symptoms. The physical examination showed a violaceus lesion soft in consistency on the left leg (Figures 1 and 2). MRI showed an oval lesion with hypointensity signal in T1 and hyperintensity in T2, with homogeneous enhance postcontrast study, and diffusion‐restriction. There was no palpable lymphadenopathy or hepatosplenomegaly. The skin lesion was biopsied and showed atypical cells (Figure 3). Immunohistochemical analysis showed cells that were positive for CD4, CD56, CD123, SPIB, TdT, and negative for MNDA (Figure 4), which supported the diagnosis of blastic plasmacytoid dendritic‐cell neoplasm (BPDCN). Complete blood count was unremarkable and peripheral blood smear did not show immature cells. Bone marrow aspirate/biopsy did not reveal the presence of neoplastic plasmacytoid dendritic cells. An ^18^(F)–FDG‐PET‐CT imaging did not reveal evidence of other lesions (Figure 5). The next‐generation sequencing (NGS) of the skin lesion biopsy specimen revealed NRAS mutation. NGS of the bone marrow biopsy specimen showed no pathogenic mutations. BPDCN is a rare, but aggressive, hematologic malignancy. The clinical features and evolution consist of two main patterns: (A) Indolent onset dominate by skin lesions followed by tumor dissemination (70–90%) and (B) Acute leukemia features with systemic involvement from the beginning (10‐30%) [1, 2]. Skin lesions can be extremely heterogeneous, but more often they are multiple and can involve any body site [2]. Typically affect older men and precede dissemination extracutaneous by a few months [1]. Confirmation of a diagnosis can pose a significant challenge in many cases, and therefore, the clinician and pathologist must have a high degree of suspicion, particularly in patients presenting with skin lesions and cytopenias. Although lack of systemic involvement at presentation may seem reassuring, survival is poor regardless of presentation [1, 2].

**FIGURE 1:**
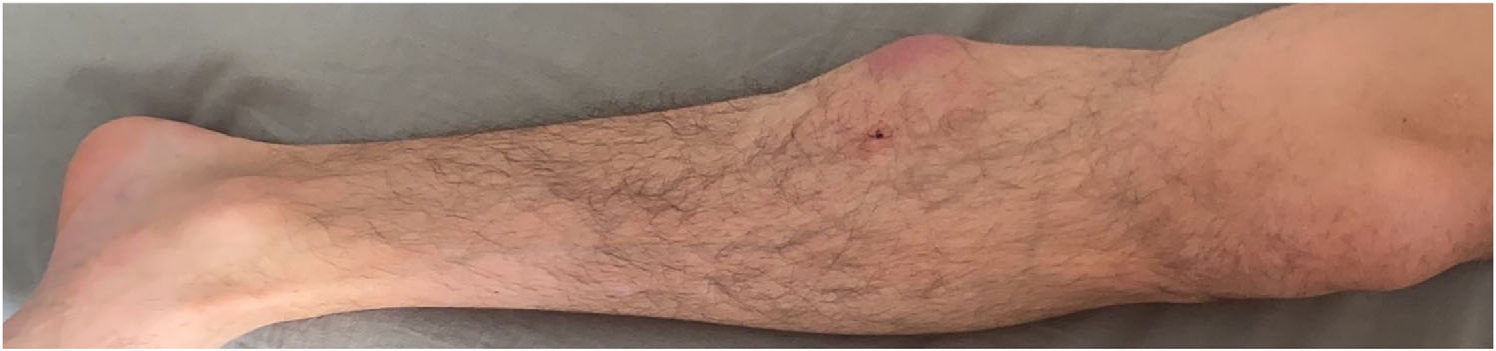
Extensive violaceus lesion on the internal side of the left leg

**FIGURE 2:**
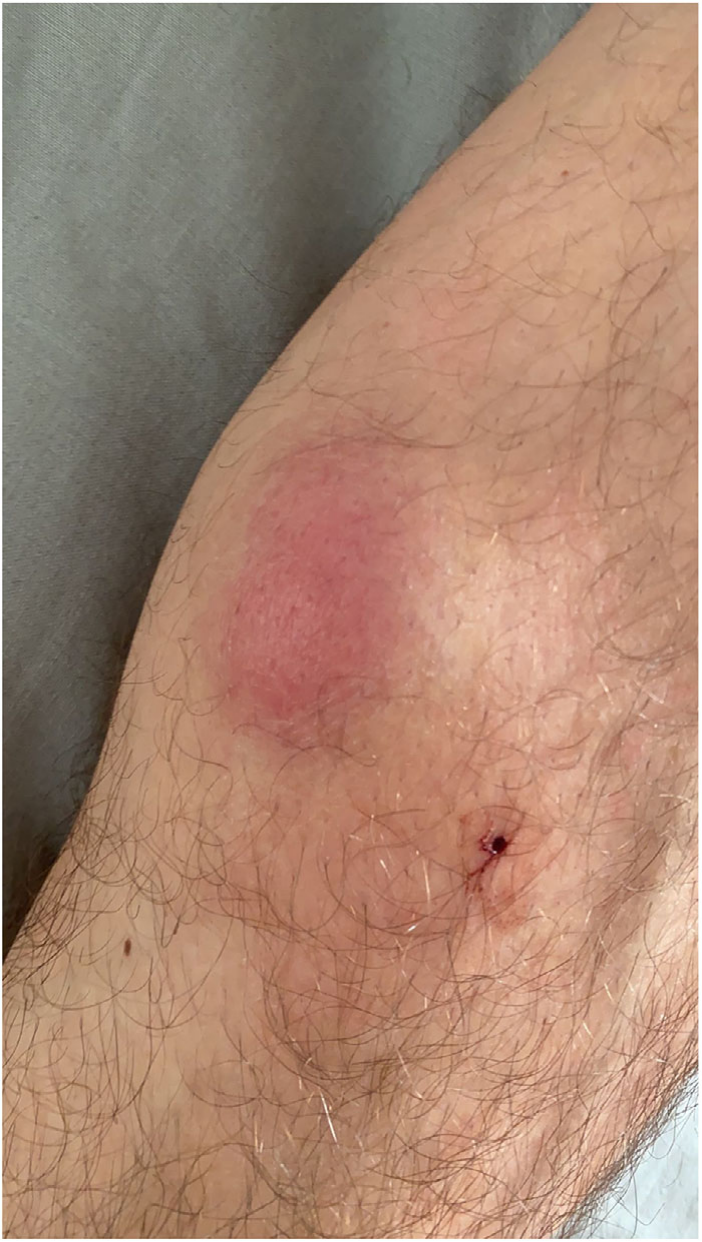
Extensive violaceus lesion on the internal side of the left leg

**FIGURE 3:**
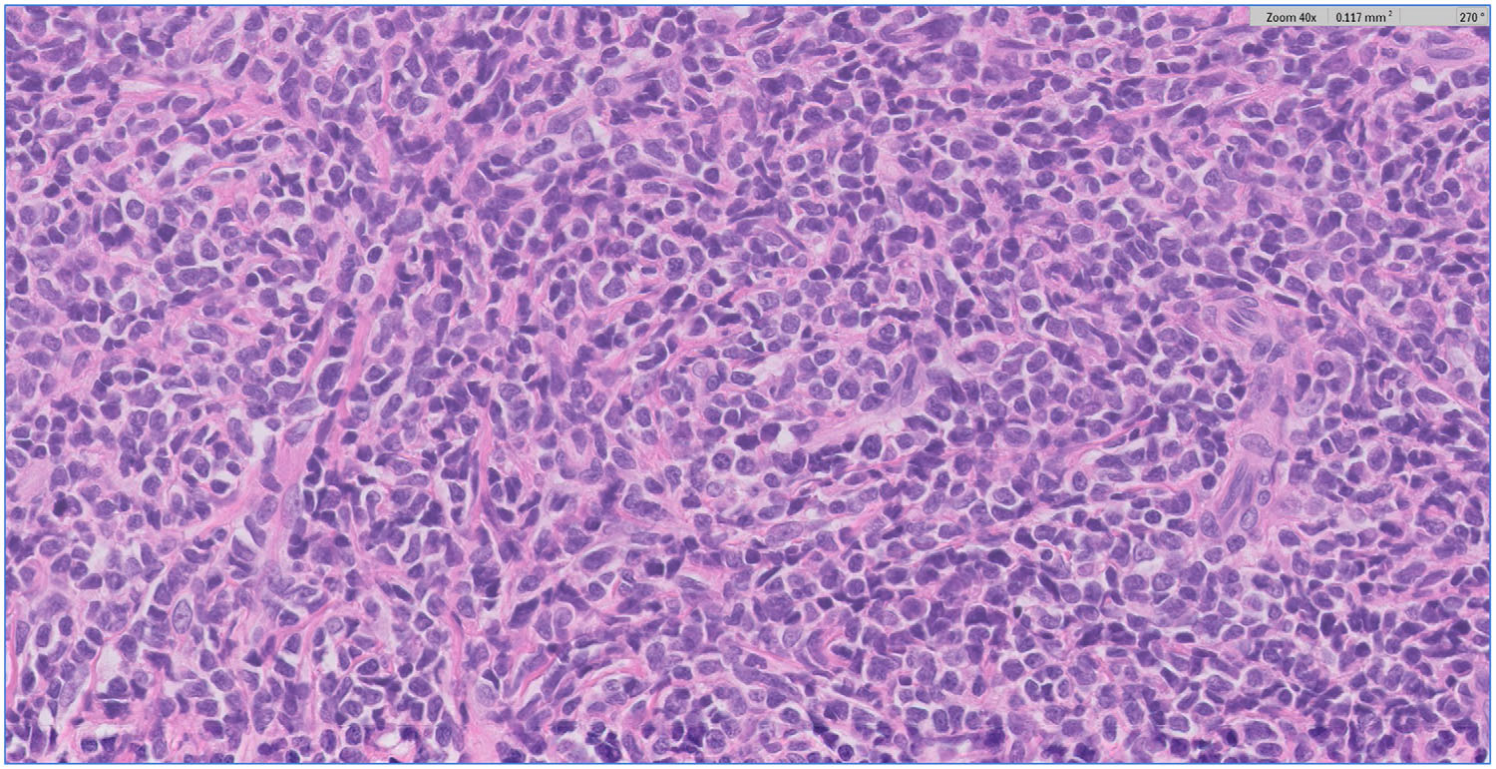
Skin lesion biopsy specimen shows atypical cells of different sizes with scant cytoplasm, cleft nucleus, irregular, hyperchromatic with occasional striking nucleolus (H&E)

**FIGURE 4:**
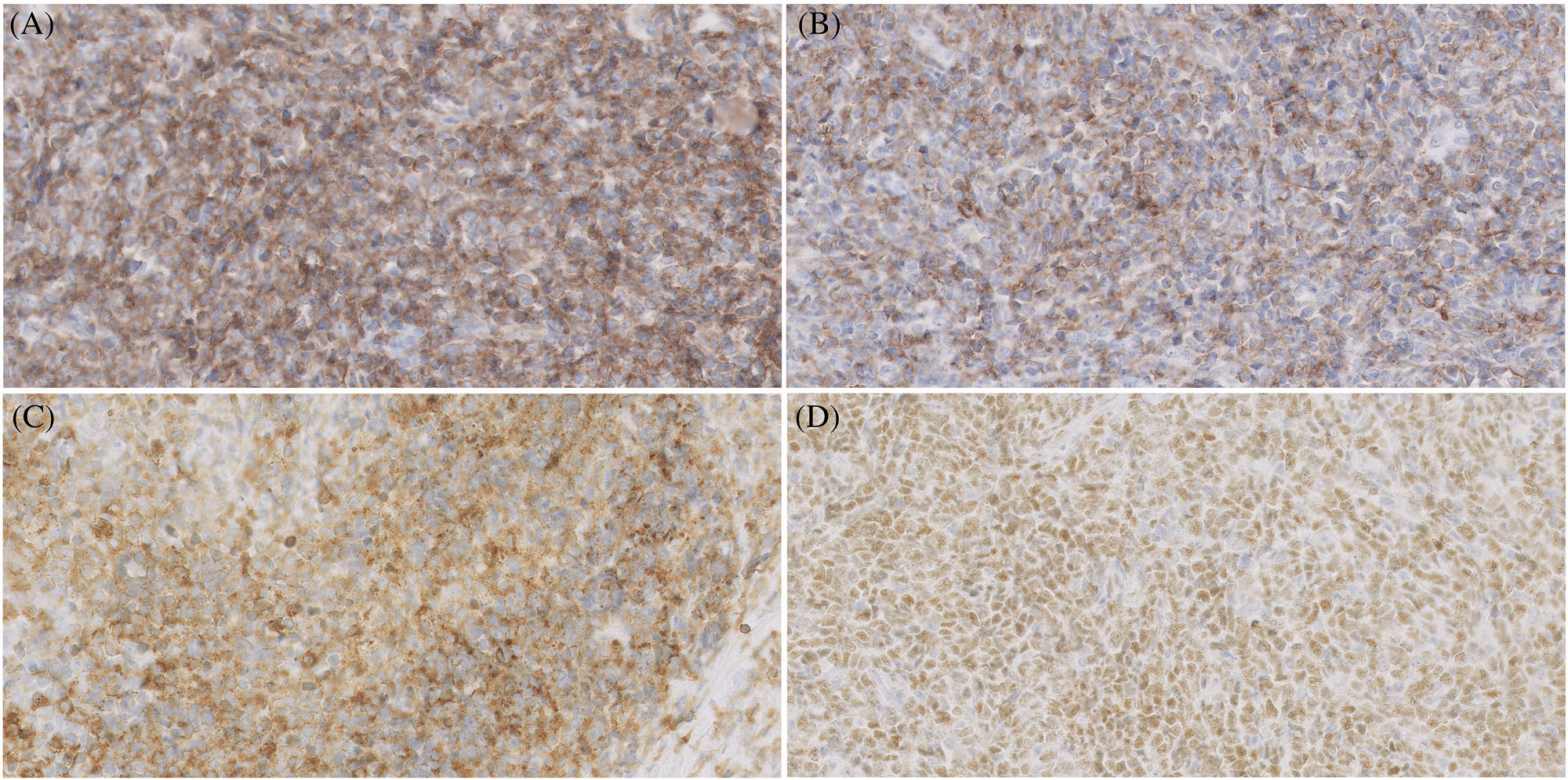
Immunostaining for CD123 (A), CD4 (B), CD56 (C), and SPIB (D) reveals clusters of cells corresponding to the malignant blast cells seen on H&E

**FIGURE 5:**
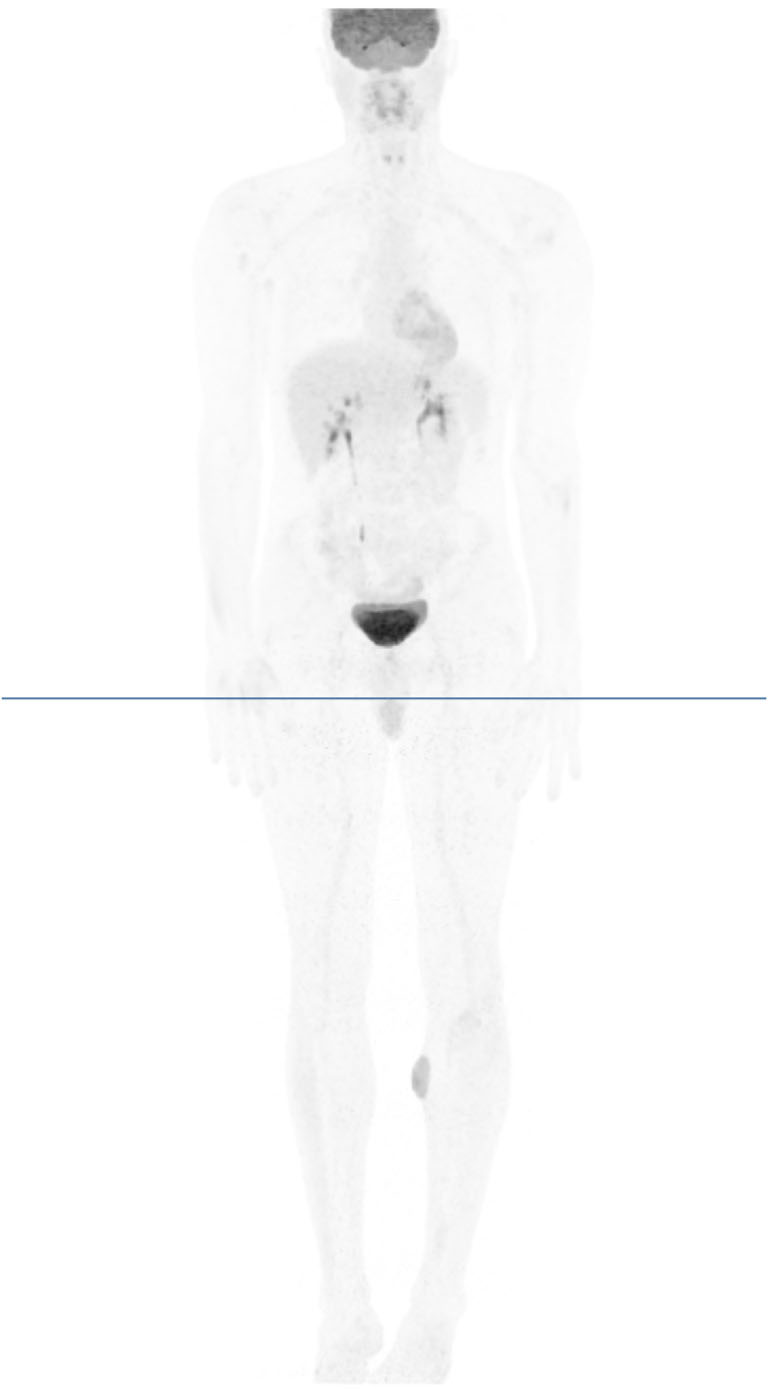
^18^(F)^–^FDG‐PET‐CT imaging show lesion with oval morphology and well‐defined borders (44 × 18 × 53 mm) with pathological uptake

## CONFLICT OF INTEREST

The authors report no conflict of interest.

## AUTHOR CONTRIBUTIONS

All authors wrote and edited the manuscript. The patient provided written informed consent for the publication of this Clinical Picture.
